# Final Analysis of COVID-19 Patients With Inflammatory Bowel Disease in Japan (J-COSMOS): A Multicenter Registry Cohort Study

**DOI:** 10.1016/j.gastha.2023.07.017

**Published:** 2023-07-31

**Authors:** Hiroshi Nakase, Yuki Hayashi, Yoshihiro Yokoyama, Takayuki Matsumoto, Minoru Matsuura, Hideki Iijima, Katsuyoshi Matsuoka, Naoki Ohmiya, Shunji Ishihara, Fumihito Hirai, Daiki Abukawa, Tadakazu Hisamatsu, J-COSMOS group, Makoto Sasaki, Masahiro Iizuka, Mikihiro Fujiya, Takayuki Matsumoto, Fukunori Kinjo, Shiro Nakamura, Noriko Kamata, Hideki Iijima, Yuri Etani, Fumiaki Ueno, Sakiko Hiraoka, Takeo Kondo, Takashi Kagaya, Makoto Naganuma, Kiyonori Kobayashi, Taku Kobayashi, Shuji Yamamoto, Yuji Naito, Tadakazu Hisamatsu, Yoki Furuta, Keichi Mitsuyama, Yu Hashimoto, Katsuhiro Arai, Shingo Kato, Itaru Iwama, Motohiro Esaki, Hiroki Tanaka, Hiroshi Nakase, Satoshi Motoya, Atsuo Maemoto, Toshifumi Ashida, Nobuaki Nishimata, Shigeaki Aono, Akira Andoh, Hironori Yamamoto, Shunji Ishihara, Toshiaki Shimizu, Maeda Yasuharu, Kenji Kinoshita, Katuyuki Fukuda, Jun Kato, Ken Takeuchi, Masakazu Nagahori, Masakatsu Fukuzawa, Masayuki Saruta, Takayoshi Suzuki, Michio Itabashi, Masaru Shinozaki, Soichiro Ishihara, Naoki Yoshimura, Katsuyoshi Matsuoka, Yoichi Kakuta, Kenichi Takahashi, Keiichi Tominaga, Ryosuke Sakemi, Sohachi Nanjo, Shusaku Yoshikawa, Keiji Ozeki, Ayako Fuchigami, Takehiko Katsurada, Kenji Watanabe, Hirotake Sakuraba, Fumihito Hirai, Takashi Hisabe, Shigeru Iwase, Naoki Ohmiya, Ryota Hokari, Katsuhiko Nakai, Takeshi Ueda, Daiki Abukawa, Shojiro Yamamoto, Kazutaka Koganei, Reiko Kunisaki, Akira Hokama

**Affiliations:** 1 Department of Gastroenterology and Hepatology, Sapporo Medical University School of Medicine, Sapporo, Japan; 2 Division of Gastroenterology, Department of Medicine, Iwate Medical University, Morioka, Japan; 3 Department of Gastroenterology and Hepatology, Kyorin University School of Medicine, Tokyo, Japan; 4 Department of Gastroenterology and Hepatology, Osaka University Graduate School of Medicine, Osaka, Japan; 5 Department of Gastroenterology and Hepatology, Toho University Sakura Medical Center, Chiba, Japan; 6 Department of Gastroenterology, Fujita Health University School of Medicine, Toyoake, Japan; 7 Department of Gastroenterology, Faculty of Medicine, Shimane University, Izumo, Japan; 8 Department of Gastroenterology and Medicine, Fukuoka University Faculty of Medicine, Fukuoka, Japan; 9 Department of Gastroenterology and Hepatology, Miyagi Children’s Hospital, Sendai, Japan

**Keywords:** Body Mass Index, Cerebrovascular Disease, Corticosteroids, SARS-CoV-2

## Abstract

**Background and Aims:**

Japan has experienced 8 waves of the coronavirus disease 2019 (COVID-19) outbreak over the past 3 years, resulting in an increasing number of deaths and incidence of severe infections. This study aimed to analyze the data from the Japanese inflammatory bowel disease (IBD) patients with COVID-19 registry (J-COSMOS) up to the eighth wave to investigate the clinical course of IBD patients with COVID-19 and factors contributing to disease severity.

**Methods:**

In this multicenter, observational, cohort study, we analyzed a cohort of 1308 IBD patients diagnosed with COVID-19, enrolled across 77 participating facilities in the J-COSMOS registry from June 2020 to December 2022. Data on age, sex, IBD (classification, treatment, and activity), and COVID-19 (symptoms, severity, and treatment) were analyzed.

**Results:**

The majority of patients (76%) were in clinical remission. According to the World Health Organization classification of COVID-19 severity, 98.4% of IBD patients had nonsevere disease, while 1.6% of patients had severe or critical disease. COVID-19 did not affect disease activity in most IBD patients. Stepwise logistic regression analysis revealed that high body mass index, and cerebrovascular disease were risk factors for severe COVID-19. Corticosteroids could affect COVID-19 severity, whereas anti-tumor necrosis factor α antibodies and thiopurines were associated with a reduced risk of severe COVID-19. No deaths were observed among IBD patients with COVID-19 registered in this cohort.

**Conclusion:**

The impact of COVID-19 on IBD disease activity and factors associated with COVID-19 severity were consistent with findings of previous reports. No deaths in Japanese patients with IBD were observed.

## Graphical abstract

**Figure:**
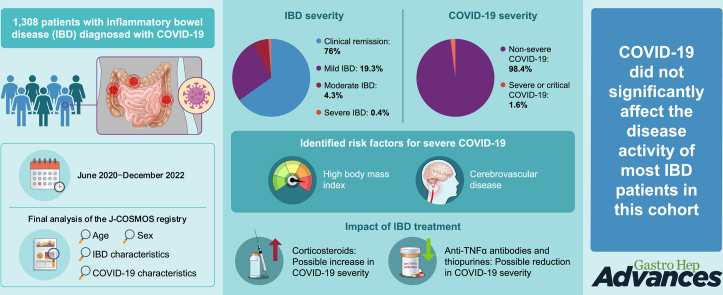

## Introduction

Coronavirus disease 2019 (COVID-19) has significantly impacted public health and healthcare systems globally.^1^^,^^2^ Mutations in severe acute respiratory syndrome coronavirus-2 (SARS-CoV-2) have also led to significant changes in the transmission mode and subsequent clinical course of the disease.^3^, ^4^, ^5^ Older adults and individuals with underlying diseases are highly likely to develop severe COVID-19 and experience exacerbation of underlying diseases following infection.^6^, ^7^, ^8^ Despite extensive research on SARS-COV-2, the future long-term impact of COVID-19 remains unknown.

Concerns regarding the impact of COVID-19 on patients with autoimmune diseases receiving immunomodulatory therapy have been reported, with therapy also possibly resulting in exacerbation of the infection.^9^, ^10^, ^11^

Inflammatory bowel disease (IBD) comprises of 2 major disease types: ulcerative colitis (UC) and Crohn's disease (CD); it has become increasingly prevalent in Japanese patients since 1950.^12^ During the COVID-19 pandemic, it is essential to understand the involvement of SARS-CoV-2 infection in the clinical course of Japanese patients with IBD, considering the differences in the severity and prognosis of patients with COVID-19 between other countries and Japan.

We have previously established a registry of Japanese IBD patients with COVID-19 infection (J-COSMOS).^13^ An interim analysis of 187 patients enrolled from June 2020 to October 2021 revealed that 7% of patients had severe COVID-19 according to the World Health Organization (WHO) criteria.^13^ Approximately 90% of the patients had experienced no change in IBD disease activity. Risk factors for severe COVID-19 were older age, higher body mass index (BMI), and steroid use.

Japan has experienced 8 waves of the COVID-19 outbreak over the past 3 years, leading to increased morbidity and mortality. Accordingly, the number of COVID-19 cases among IBD patients has also increased. Therefore, this study aimed to reanalyze the registry data up to the eighth wave and to investigate the clinical course of IBD patients infected with COVID-19 and the factors contributing to disease severity.

## Materials and Methods

This multicenter, registry cohort study was conducted by the research study group on intractable inflammatory bowel disorder of the Ministry of Health, Labor and Welfare in Japan using information obtained from eligible patients. No new intervention was implemented for this study. The protocol of this study was approved by the institutional review boards at each institution and registered publicly on the University Hospital Medical Information Network (registration number UMIN000040656).^13^

### Patients

Patients who were diagnosed with IBD and tested positive for COVID-19, either in the outpatient clinic or in the participating institutions, from June 2020 to December 2022 were eligible to be included in the registry. The following IBD types were diagnosed: UC, CD, IBD-unclassified, and intestinal Behçet's disease (BD). A confirmed diagnosis of COVID-19 was defined as the presence of the SARS-CoV-2 genome, confirmed by real-time polymerase chain reaction findings, the positivity of the antigen test for SARS-CoV-2 using nasopharyngeal swab or saliva samples, or positive serum antibodies against SARS-CoV-2.^14^, ^15^, ^16^ Asymptomatic SARS-CoV-2 carriers were also included in the registry. After January 24, 2022, this registry also included deemed-positive cases in accordance with the definition provided by the Ministry of Health, Labor and Welfare. Deemed-positive cases were defined as the cases wherein patients developed COVID-19 like symptoms after living with or coming into contact with a confirmed positive patient and required COVID-19 treatment without undergoing a SARS-CoV-2 test. For patient who were presumed to be positive for the disease, the date of COVID-19 diagnosis was used as the estimated date of onset. IBD patients who were hospitalized or had undergone outpatient treatment for COVID-19 were registered. Patients who refused to participate in this study were excluded.

### Survey Method

Physicians completed the survey by reviewing the medical records available at each institution. The obtained data were anonymized and recorded in a password-locked Excel file entitled “Case Report Form.” The file was then e-mailed to the person in charge at the Department of Gastroenterology, Sapporo Medical University (Sapporo, Japan). An electronic data capture system for registration of clinical information, which was established at Sapporo Medical University, has been used since June 2021.

### Survey Items

The following data were recorded and analyzed: 1) Medical history: age, gender, height, weight, IBD diagnosis, smoking status, and comorbidities (cardiovascular disease, diabetes, asthma, chronic respiratory disease, hypertension, malignancy, cerebrovascular disease, chronic renal disease, chronic liver disease, and others), 2) Disease activity (“active” status was defined by a partial Mayo (pMayo) score ≧3 with a rectal bleeding subscore ≧1 for UC^17^^,^^18^; and a Harvey-Bradshaw Index (HBI) ≧5 for CD^19^; and a subjective judgment of the attending physician for IBD-U, and intestinal BD, duration of disease, disease type, treatment (5-aminosalicylic acid, thiopurines, steroids, calcineurin inhibitors, biologics, Janus kinase (JAK) inhibitors, nutritional therapy, and cytapheresis), exacerbation of IBD, and changes in IBD treatment during COVID-19, 3) Information on COVID-19: date of diagnosis, number of days from onset to diagnosis, testing methods that led to diagnosis (PCR, antibody, or other), signs and symptoms of COVID-19 (fever, cough, dyspnea, pharyngitis, diarrhea, arthralgia-myalgia/asthenia, rhinitis, dysosmia, dysgeusia, and dysphonia), presence of pneumonia, and COVID-19 treatment and severity/outcomes (outpatient treatment, inpatient treatment, intensive care, and death). The severity of COVID-19 was determined based on the WHO classification.^20^ The time period “Before” was defined as the time immediately before SARS-COV-2 infection; the time period “During” was defined as the time when patients’ symptoms were most severe; and the time period “After” was defined as the time when the acute phase of COVID-19 symptoms had improved (approximately 1–2 weeks after the onset).

### Statistical Analysis

Microsoft Excel 16.0 (Microsoft Corp., Redmond, WA) was used to record patients’ data and analyze the clinical background factors and disease outcome. To address the issue of missing values in this cohort, we undertook the following measures: (1) We created the electronic data capture software with the aim of preventing the entry of invalid values and preventing missing values by ensuring that no survey items were ignored, (2) We removed missing values in the observations using pairwise deletion, (3) In the logistic regression analysis, if more than 5% of the data were deleted, we used the multiple imputation method to assign missing values.

Analyses that require specific tests were performed using EZR software (Saitama Medical Center, Jichi Medical University, Saitama, Japan).^21^ Nominal variables were analyzed using Fisher exact test. Odds ratio (OR) was calculated at 95% confidence intervals (CIs). A t-test with 95% CIl was performed to analyze the means data. The relationship between COVID-19 severity and risk factors was analyzed using stepwise logistic regression analysis (Akaike’s Information Criterion [AIC], backward/forward). An alluvial plot was used to describe the repeated measurements of IBD activity during COVID-19. To identify risk factors for worsening IBD activity, multiple regression analysis or a stepwise regression analysis using the AIC method was performed on patients who had a pMayo or HBI change greater >0. Items with a *P* value < .3 were used as explanatory variables in the ordinal logistic regression analysis and stepwise method using the AIC method.

## Results

### Patients’ Characteristics

We note an increase in the COVID-19 registration over the past year. At the time of the interim analysis (October 31, 2021), the registered number of Japanese patients with COVID-19 was 1,718,417. However, at the time of this study analysis (November 30, 2022), the number was 24,793,166. Thus, the number of Japanese patients with COVID-19 increased by 14.4-fold in 1 year. This is reflected in the increase in the number of registered patients included in this analysis.

**Figure 1:**
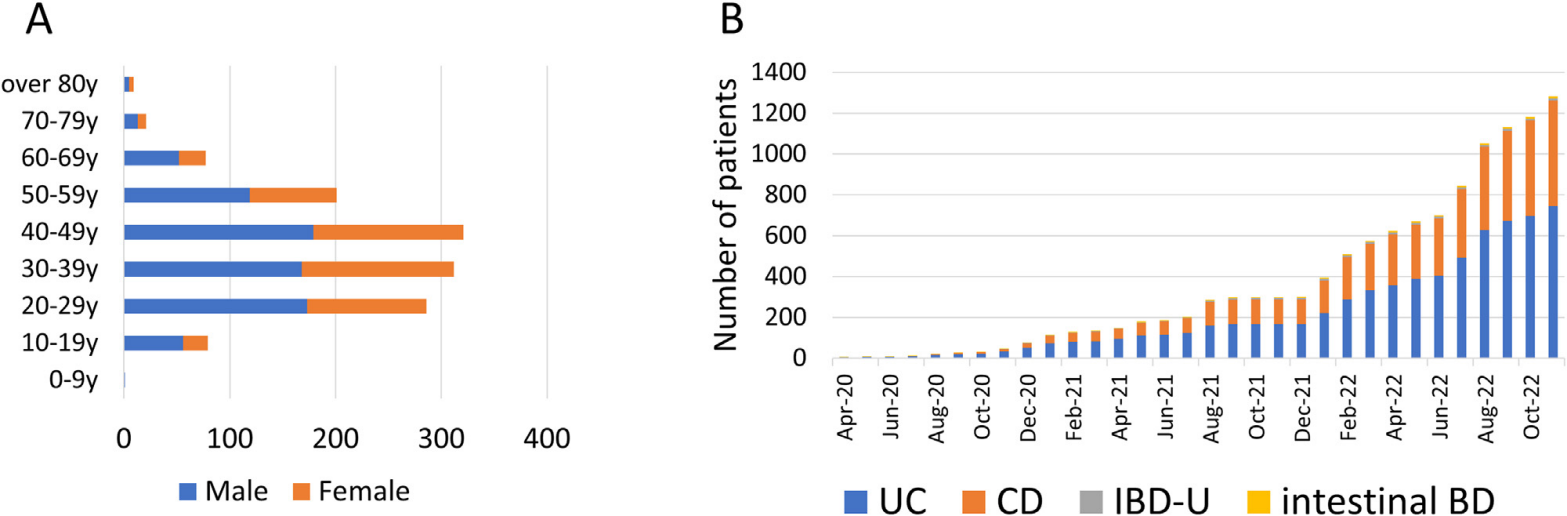
(A) Age distribution of Japanese IBD patients with COVID-19 (B) Transition of the number of Japanese IBD patients with COVID-19.

A total of 1308 IBD patients with COVID-19 were registered between June 2020 and December 2022. Their mean age was 39 years, and the maximum number of registered patients belonged to the age groups of 30−39 and 40−49 years (Figure 1A). The patients had a diagnosis of UC (n = 766), CD (n = 523), IBD-U(n = 10), and BD (n = 9). The number of females was 542 (41.4%) and 26.4 % of the registered patients had existing comorbidities. In total, 76% (940/1236) of patients were in clinical remission, 19% (238/1236) had mildly active disease, 4.3% (53/1236) had moderately active disease, and 0.4% (5/1236) had severely active disease at the time of COVID-19 diagnosis. The baseline characteristics of the patients are summarized in Table 1.

**Table 1:** Baseline Characteristics of Patients

Participating facilities (n)	77
Registered cases (n)	1308
Age (y) ±SD	39 ± 14.2
Gender (M/F) (n)	766/542
Height (cm) ± SD	166.0 ± 10.2
Weight (kg) ± SD	59.0 ± 23.2
BMI (kg/m^2^) ± SD	21.5 ± 11.9
BMI > 30 kg/m^2^ (n)	55
Smoker (current) (%)	112/1229 (9.1 %)
Past smoking (%)	241/1229 (19.6 %)
All comorbidity (%)	330/1248 (26.4 %)
Diagnosis of IBD	
UC	766
CD	523
IBD-U	10
Intestinal BD	9
Duration of IBD (mo) ± SD	105 ± 106.6
Disease activity of IBD (at diagnosis of COVID-19)	
Remission	940/1236 (76.0%)
Mild	238/1236 (19.3%)
Moderate	53/1236 (4.3%)
Severe	5/1236 (0.4%)

UC, Ulcerative Colitis; CD, Crohn’s Disease; BMI, Body Mass Index; IBD-U, Inflammatory Bowel Disease Unclassified; Intestinal BD, Intestinal Behçet's Disease; COVID-19, Coronavirus Disease 2109.

### Transition of the Number of IBD Patients With COVID-19 in Japan and the Vaccinated Population

The number of IBD patients with COVID-19 in Japan has gradually increased. This upward trend was observed until the eighth wave of the pandemic (from August 2021 to December 2022) (Figure 1B). As vaccination was prioritized for older individuals in Japan in April 2021, the increase in the number of registered patients aged > 60 years was gradual. Meanwhile, the maximum increase was observed in the number of patients in the age group of 20–50 years (Figure A1).

### IBD Treatment

**Figure 2:**
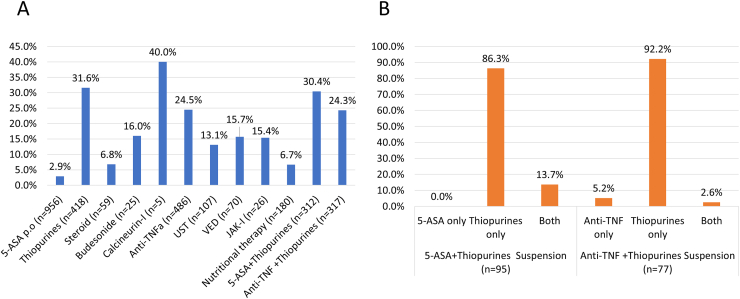
(A) Proportion of patient who have discontinued each IBD therapy; (B) Left figure; of the 312 patients receiving both 5-ASA and thiopurines, 95 patients (30.4%) discontinued any of the drugs. Proportion of these patients receiving both 5-ASA and thiopurines who have discontinued either, or both. 5-ASA only: 0% of patients discontinued 5-ASA alone, thiopurines only: 86.3% of patients discontinued thiopurines alone, both: 13.7% of patients discontinued both. Right figure; of the 317 patients receiving both anti-TNFα antibodies and thiopurines, 77 patients (24.3%) discontinued any of the drugs. Proportion of these patients receiving both anti-TNFα antibodies and thiopurines who discontinued either or both. anti-TNFs: 5.2% of patients have discontinued anti-TNFα antibodies alone, thiopurines only: 92.2% of patients discontinued thiopurines alone, both: 2.6% of patients discontinued both. 5-ASA, 5-aminosalicylic acid.

Of the 1308 registered patients, 73% (956/1308) were being treated with 5-aminosalicylic acid (5-ASA), 37.1% (486/1308) with anti-tumor necrosis factor (TNF) α antibodies, 8.2% (107/1308) with ustekinumab, 5.4% (70/1308) with vedolizumab, 2.0% (26/1308) with JAK inhibitors, 32% (418/1308) with thiopurines, 4.5% (59/1308) with steroids,1.9% (25/1308) with budesonide, and 0.4% (5/1308) with calcineurin inhibitors (Figure A2). Calcineurin inhibitors were discontinued by 40%, thiopurines by 31.6%, anti-TNFα antibodies by 24.5%, vedolizumab by 15.7%, JAK inhibitors by 15.4%, and ustekinumab by 13.1% of patients after the COVID-19 diagnosis (Figure 2A). The number of enrolled patients (84) undergoing treatment with steroids including budesonide was low, with only 6.8% of patients discontinuing its use. Among patients receiving both thiopurines and anti-TNFα antibodies, 92.2% of patients discontinued thiopurines alone, 5.2% discontinued anti-TNFα antibodies alone, and 2.6% discontinued both. Among patients receiving both 5-ASA and thiopurines, 86.3% of patients discontinued thiopurines alone (Figure 2B).

### COVID-19 Symptoms, Severity and Factors Related to Clinical Outcomes

According to the WHO classification of COVID-19 severity, 98.4% (1257/1278) of IBD patients had nonsevere disease, while 1.6% (21/1278) of patients had severe or critical disease (Table 2). The median age (Interquartile range: [IQR]) of IBD patients with severe COVID-19 was significantly higher than that of patients with non-severe disease (58 [45–60] vs 38 [28–48] years, *P* < .001). In addition, the BMI (IQR) of IBD patients with severe COVID-19 was significantly higher than that of the patients with nonsevere disease (23.7 [21.8–26.8] vs 21.5 [19.5–23.9] kg/m^2^, *P* = .0033). IBD patients with severe COVID-19 demonstrated significantly higher rates of comorbidities than those with non-severe COVID-19 (OR = 9.38). Fisher's exact test demonstrated significant differences in the prevalence other respiratory illnesses (OR = 19.6), hypertension (OR = 8.95), and cerebrovascular diseases (OR = 39.0) between patients with severe COVID-19 and non-severe COVID-19 (Table 2). Patients with severe COVID-19 experienced pneumonia and respiratory symptoms, followed by dysgeusia, which is consistent with the findings of existing reports (Table A1). Examination of the trends in the proportion of IBD patients with severe and critical COVID-19, stratified by the approximate timing of the outbreak of the mutant strain of SARS-COV-2, revealed a gradual decline in the number of individuals with severe disease. No registered patients died during the observation period (Table 3). Stepwise logistic regression analysis revealed that high BMI (OR = 1.20), and cerebrovascular disease (OR = 1.14) were risk factors for COVID-19 (Table A2).

**Table 2:** Patients’ Background Characteristics Stratified by Severity of COVID-19

Factor	Non-severe	Severe	Fisher-test *P* value	Fisher-test Odds
Age (y) [IQR]	38 [28–48]	58 [45–60]	<.001	-
Gender M/F	729/528	15/6	.27	0.55
BMI (kg/m^2^) [IQR]	21.5 [19.5–23.9]	23.7 [21.8–26.8]	.0033	-
History% (n)				
DM	2.5% (30)	9.5% (2)	.10	4.08
CKD	1.3% (16)	0% (0)	1	0
Liver diseases	4.8% (57)	4.8% (1)	1	1.0
Asthma	5.0% (59)	9.5% (2)	.29	2.02
COPD	0.1% (1)	0% (0)	1	0
Other respiratory illness	0.8% (10)	14.3% (3)	<.001	19.6
Cardiovascular diseases	2.2% (26)	4.8% (1)	.38	2.25
HT	5.3% (63)	33.3% (7)	<.001	8.95
Cerebrovascular diseases	0.4% (5)	14.3% (3)	<.001	39.0
Malignancy	1.3% (16)	0% (0)	1	0
All	25.4% (304)	76.2% (16)	<.001	9.38
Smoker% (n)				
Never	70.1% (784)	52.4% (11)	.159	-
Current	9.5% (106)	14.3% (3)		
Past smoking	20.4% (228)	33.3% (7)		
Diagnosis of IBD				
UC	738	17	.25	-
CD	501	4		
IBD-U	10	0		
Intestinal BD	8	0		
Clinical phenotype				
UC				
Proctitis	151	5	.28	-
Left sided	196	4		
Pancolitis	368	6		
Segmental	13	1		
CD				
Ileitis	107	1	.40	-
Colitis	58	1		
Ileo-colitis	346	2		
Isolated upper	1	0		
Disease activity (at diagnosis of COVID-19)				
Remission	922	18	.12	-
Mild	236	2		
Moderate	53	0		
Severe	4	1		
Treatment of IBD % (n)				
5-ASA p.o	73.2% (899)	89.5% (17)	.19	3.11
Thiopurines	24.8% (279)	5.0% (1)	.038	0.16
Steroid	4.2% (53)	10.0% (2)	.21	2.51
Budesonide	1.8% (22)	0% (0)	1	0
Calcineurin-I	0.2% (3)	0% (0)	1	0
Anti-TNFα	31.9% (365)	0% (0)	<.001	0
UST	7.5% (93)	0% (0)	.40	0
VED	4.8% (60)	0% (0)	.62	0
JAK-I	1.8% (22)	0% (0)	1	0.00
Diagnosis of IBD				
UC	738	17	.25	-
CD	501	4		
IBD-U	10	0		
Intestinal BD	8	0		
Clinical phenotype				
UC				
Proctitis	151	5	.28	-
Left sided	196	4		
Pancolitis	368	6		
Segmental	13	1		
CD				
Ileitis	107	1	.40	-
Colitis	58	1		
Ileo-colitis	346	2		
Isolated upper	1	0		
Disease activity (at diagnosis of COVID-19)				
Remission	922	18	.12	-
Mild	236	2		
Moderate	53	0		
Severe	4	1		
Treatment of IBD % (n)				
5-ASA p.o	73.2% (899)	89.5% (17)	.19	3.11
Thiopurines	24.8% (279)	5.0% (1)	.038	0.16
Steroid	4.2% (53)	10.0% (2)	.21	2.51
Budesonide	1.8% (22)	0% (0)	1	0
Calcineurin-I	0.2% (3)	0% (0)	1	0
Anti-TNFα	31.9% (365)	0% (0)	<.001	0
UST	7.5% (93)	0% (0)	.40	0
VED	4.8% (60)	0% (0)	.62	0
JAK-I	1.8% (22)	0% (0)	1	0.00

BMI, body mass index; Calcineurin-I, calcineurin inhibitors; CD, Crohn’s disease; COPD, chronic obstructive pulmonary disease; COVID-19, coronavirus disease 2019; DM, diabetes mellitus; HT, hypertension; IBD-U, inflammatory bowel disease unclassified; intestinal BD, intestinal Behçet's disease; JAK-I, Janus-kinase inhibitors; UC, ulcerative colitis; UST, ustekinumab; VED, vedolizumab; 5-ASA, 5-aminosalicylic acid.

**Table 3:** Trends in the Proportion of IBD Patients With Severe and Critical COVID-19 Stratified by the Approximate Timing of the Outbreak of the Mutant Strain of SARS-CoV-2

COVID-19 severity	Total	Before delta variant	Delta variant
Nonsevere	1257	222	46
Severe	18	13	3
Critical (intensive care unit or ventilation)	3	3	0
Death	0	0	0

The rates of severe COVID-19 in patients with UC and CD were 2.3% (17/755) and 0.8% (4/505), respectively, although no significant difference was noted between the 2 groups. Neither the clinical phenotype nor disease activity was associated with COVID-19 severity for both UC and CD. Among the IBD treatment modalities, corticosteroids could affect COVID-19 severity (*P* = .21, OR = 2.51, 95% CI [0.276–10.9]), whereas anti-TNFα antibodies (*P* < .001, OR = 0) and thiopurines (*P* = .038, OR = 0.16) were associated with a reduced risk of severe COVID-19. However, ustekinumab, vedolizumab, and JAK-I did not affect COVID-19 severity (Table 2).

### Changes in IBD Activity During COVID-19

**Figure 3:**
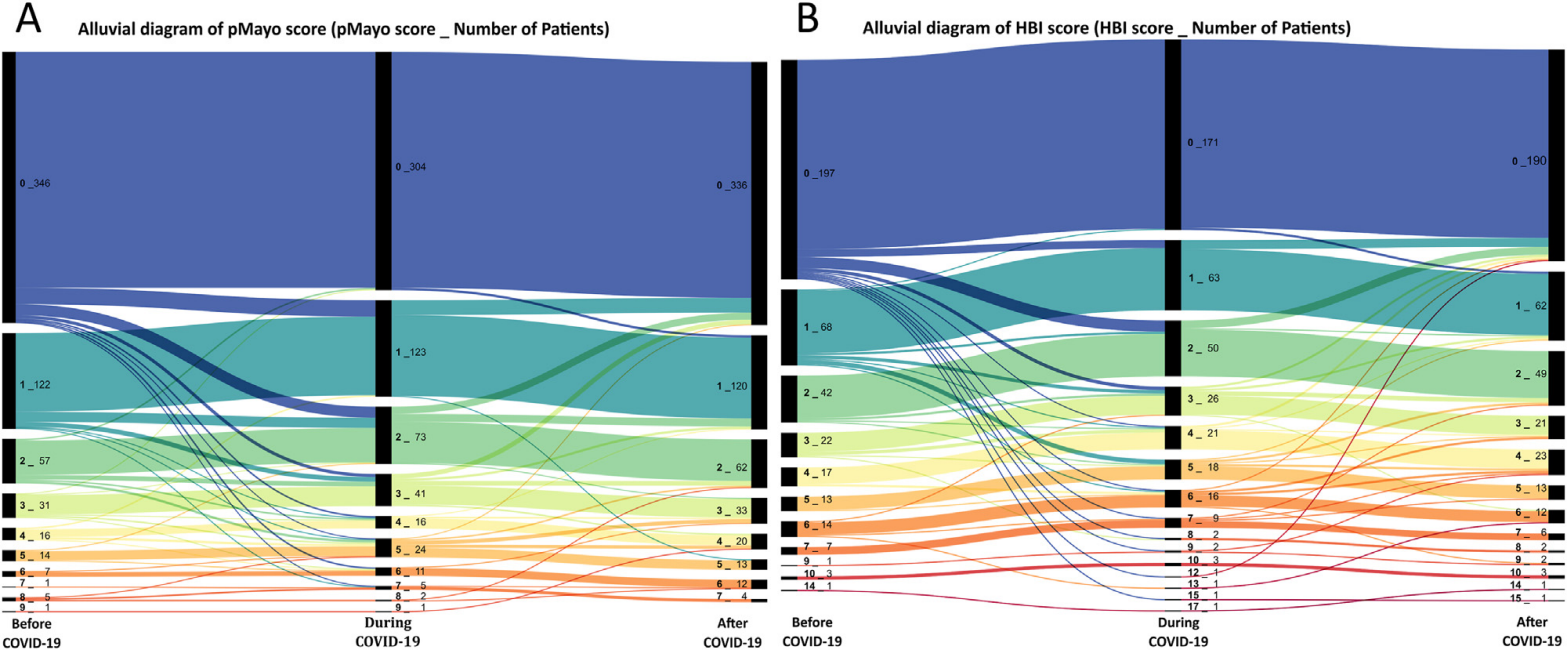
Alluvial diagram of change in disease activity: (A) changes in pMayo score before, during, and after COVID-19 (B) changes in HBI score before, during, and after COVID-19 (A and B).

The alluvial diagram demonstrates the changes in pMayo score and HBI before, during, and after COVID-19, which revealed a slight change in the activity of UC and CD during the observation period (Figure 3A and B). Changes in IBD activity were evaluated by the difference of pMayo score for UC and by HBI for CD before, during, and after COVID-19. The stratification of changes in Mayo score and HBI score demonstrated that most IBD patients experienced no change in disease activity during and after recovery from COVID-19. Regarding changes in disease activity score before and after infection, the number of UC and CD patients with a score of 1 or higher decreased, while the number of those with a score of 0 or lower increased (Figure A3A and B). Ordinal logistics regression analysis demonstrated that patients receiving 5-ASA had significantly fewer CD flares during COVID-19 (Table 4).

**Table 4:** Association Between IBD Drugs and Disease Activity Before and After COVID-19

UC	During–Before			After–Before		
	Odds ratio	95% CI	*P* value	Odds ratio	95% CI	*P* value
5-ASA	1.51	[0.77–3.26]	.26	1.31	[0.56–3.60]	.57
Thiopurine	1.31	[0.72–2.31]	.37	1.31	[0.58–2.73]	.49
Steroid	1.59	[0.44–4.54]	.42	2.20	[0.04–0.93]	.26
Ant-TNF-α	0.53	[0.21–1.15]	.14	0.272	[0.49–7.18]	.08

CD	During–Before			After–Before		
	Odds ratio	95% CI	*P*-value	Odds ratio	95% CI	*P*-value
5-ASA	0.31	[0.15–0.64]	.0016^a^	0.24	[0.079–0.67]	.0082^a^
Thiopurine	1.38	[0.62–2.94]	.42	2.10	[0.72–6.0]	.16
Ant-TNF-α	0.54	[0.26–1.10]	.089	0.69	[0.25–1.9]	.47

Ordinal logistic regression analysis was performed on patients with a change in partial Mayo (pMayo) score or Harvey-Bradshaw Index (HBI) of > 0. Items with a *P*-value < .3 were used as explanatory variables in either multiple regression analysis or a stepwise method using the Akaike’s Information Criterion (AIC) method.

IBD, inflammatory bowel disease; COVID-19, coronavirus disease 2019.

a *P* < .05.

## Discussion

This final survey demonstrated the characteristics and clinical outcome of COVID-19 in Japanese IBD patients. More male patients were registered than female patients, but sex was not associated with COVID-19 severity. In total, 76% of the patients diagnosed with COVID-19 were in remission. Only 58 patients had moderately or severely active disease. However, there was no association between disease activity and COVID-19 severity. Most patients with UC and CD did not experience worsening of disease activity due to COVID-19. Among the different IBD treatments, steroid use alone was associated with increased COVID-19 severity (OR = 2.51). Stepwise logistic regression analysis revealed that higher BMI and cerebrovascular disease were risk factors for COVID-19 severity. A critical finding from the survey is that no deaths were reported among the enrolled IBD patients.

Among the IBD patients, 98.4% had non-severe COVID-19, while 1.6% of patients had severe or critical disease. In the interim analysis, 7% of patients had severe COVID-19.^13^ However, this proportion decreased in this final analysis. Hence, these findings indicate the association between COVID-19 variants and reduced disease severity, in accordance with the trends observed in the general population. Surveillance epidemiology of coronavirus under research (SECURE-IBD) and a systematic review have demonstrated the proportion of patients corresponding with severe COVID-19 was 7%–11.4%.^22^^,^^23^ However, as the data were only available until 2021, we could not directly compare these with the present data. Considering the global decline in the severity of COVID-19, COVID-19 severity in IBD patients is also expected to decrease.

No deaths were reported among IBD patients with COVID-19 in Japan, who were registered in this cohort. The possible reasons for this include: (1) prioritizing vaccination for older individuals in Japan in April 2021, which could reduce mortality as higher age is a risk factor of COVID-19 severity; (2) according to the data from Japan COVID-19 Survey and the Questionnaire for Inflammatory Bowel Disease (J-DESIRE), Japanese IBD patients received regular medical care to prevent disease flare ups, leading to improved disease activity status, and reduced COVID-19 severity^12^; (3) corticosteroids use was associated with COVID-19 severity.^13^^,^^22^^,^^23^ Hence, the low rate of steroid use amongst the enrolled patients could have also contributed to the lack of deaths. Additionally, even when the physicians encountered patients with active disease who needed steroid therapy, the steroid dose was decreased to reduce the risk of severe COVID-19, according to the recommendation by Japan Taskforce consensus.^24^

This survey also focused on the change in disease activity in IBD patients with COVID-19. As also reported in the interim analysis of J-COSMOS^13^ and other reports,^25^ COVID-19 did not affect disease activity in most IBD patients. Multivariable analysis of SECURE-IBD data demonstrated that the use of 5-ASA/sulfasalazine use was positively associated with adverse COVID-19 outcomes in IBD patients.^22^ We also observed that 5-ASA could be associated with COVID-19 severity (OR = 3.11), despite no significant differences. Ordinal logistics regression analysis demonstrated that CD patients receiving 5-ASA experienced significantly fewer flare ups during COVID-19 infection. The association of 5-ASA with CD flare up prevention could be attributed to several reasons. We have already reported that the impaired tryptophan metabolism in patients with severe COVID-19 is associated with decreased expression of *ACE2, AHR, CARD9* in the ileal mucosa. ACE2 and AhR regulate tryptophan absorption in the small intestine and modulate the intestinal microbiota by enhancing the expression of antimicrobial peptides.^26^ 5-ASA is reported to up-regulate the AhR target gene Cyp1A1.^27^ Hence, it can be speculated that in CD, 5-ASA acted on the small intestinal mucosa and triggered the production of antimicrobial peptides which stabilized intestinal bacteria. Therefore, this medication could also have induced the induction of regulatory T cells, which would have contributed to the prevention of relapse, even when accompanied by COVID-19. In addition, as 5-ASA is not likely to be used as maintenance therapy for CD patients overseas, SECURE-IBD could not evaluate the effect of 5-ASA on COVID-19-infected CD patients in detail 22. In the future, we need to elucidate the exact mechanism of 5-ASA in preventing flare ups in CD patients infected with COVID-19.

According to the previous interim analysis, the number of patients receiving advanced therapy, other than anti-TNFα antibody agents was small; therefore, the risk of COVID-19 severity for patients prescribed such agents could not be examined in detail.^13^ This survey revealed that none of the patients receiving anti-TNFα antibodies, other biologic agents, or JAK inhibitors experienced severe COVID-19. We observed that anti-TNFα antibodies and thiopurines were associated with a reduced risk of severe COVID-19. These results suggest the importance of using these drugs to maintain steroid-free remission in IBD patients and to reduce COVID-19 severity. These results are also consistent with the findings of several studies, which suggest that advanced therapies, such as anti-TNFα antibodies and JAK inhibitors, could suppress the COVID-19-induced cytokine storm and prevent severe disease.^22^^,^^23^

The interim analysis of J-COSMOS demonstrated that older age, BMI, presence of comorbidities, and corticosteroid use were associated with increased severity of COVID-19 in accordance with the SECURE-IBD findings.^22^ However, in this final survey, multivariable analysis demonstrated that BMI and history of cerebrovascular diseases were risk factors for COVID-19 severity. These results are consistent with the risk factors reported for the general population, and no IBD-specific risk factors were observed. It is not as yet clear why age was not identified as a risk factor; however, this could be due to the variation in viral strains and the low number of severe COVID-19 cases included in this cohort.

This study has several limitations. First, as reported in the interim analysis, not all IBD patients with COVID-19 in Japan were included due to the lack of a national registry for IBD patients. However, the data included in this study are reliable because all patients were registered from facilities with IBD specialists. Second, the number of registered patients aged <10 years was smaller than the number of patients aged <10 years in Japan as a whole, which could result in a potential bias. Third, since this study was exploratory in design, aiming to investigate an unknown infectious disease, we performed multiple subanalyses in a multiple hypothesis testing situation in addition to analyzing the primary endpoint; therefore, this study could be considered prone to alpha error.

## Conclusion

We have reported the final analysis of the COVID-19 registry in Japanese IBD patients. No deaths were reported among the IBD patients with COVID-19 registered in this cohort. Most IBD patients had nonsevere COVID-19 and did not experience worsening of disease activity due to COVID-19. Advanced IBD therapy also did not affect the severity of COVID-19. These results indicate that during the COVID-19 pandemic, appropriate management of IBD patients led to “zero” deaths in this cohort.

## Supplementary Materials

Figures A1–A3 and Tables A1–A2

Supplementary Figures JCOSMOS
