# Supplementary material for: Final Analysis of COVID-19 Patients With Inflammatory Bowel Disease in Japan (J-COSMOS): A Multicenter Registry Cohort Study
Source: Gastro Hep Adv. 2023 Jul 31;2(8):1056–65. doi: 10.1016/j.gastha.2023.07.017 (PMC11307685; doi:10.1016/j.gastha.2023.07.017)
Supplement: Figures A1–A3 and Tables A1–A2 [file mmc1.docx]

Supplementary Figure 1. Transition of the number of Japanese inflammatory bowel disease (IBD) with COVID-19 stratified by age

Supplementary Figure 2. Treatment of Japanese inflammatory bowel disease (IBD) at COVID-19 diagnosis. The blue bar indicates treatment continuation, an orange bar indicates treatment discontinuation, and a gray bar indicates additional treatment

5-ASA, 5-aminosalicylic acid; anti-TNF a, anti-TNFα antibodies; Calcineurin-I, calcineurin inhibitors; UST, ustekinumab; VED, vedolizumab; JAK-I, Janus-kinase inhibitors; GMA, apheresis therapy

Supplementary Figure 3. (A) Changes in disease activity during-before and after-before in inflammatory bowel disease (IBD) patients with COVID-19 evaluated by the partial Mayo (pMayo) score for ulcerative colitis (UC) and (B) by Harvey-Bradshaw Index (HBI) score for Crohn’s disease (CD)

“During-before” means the difference between the worst score of disease activity during COVID-19 and the score immediately before COVID-19. “After-before” means the difference between the score of disease activity after the end of COVID-19 and the score immediately before COVID-19

**Supplementary Table 1. Symptoms stratified by severity of COVID-19.**

| **Symptom** % (n) | **Non-severe** | **Severe** | **p-value (Fisher-test)** | **Odds (Fisher-test)** |
| --- | --- | --- | --- | --- |
| Fever (≧37.5℃) | 85.1% (1047) | 95.0% (19) | 0.341 | 3.32 |
| Respiratory symptoms | 61.1% (753) | 90.5% (19) | 0.0055 | 6.05 |
| Pneumonia | 4.9% (45) | 95.2% (20) | <0.001 | 383.8 |
| Headache | 34.7% (421) | 33.3% (6) | 1 | 2.19 |
| Fatigue | 49.5% (603) | 30.0% (6) | 0.075 | 2.37 |
| Vomit | 3.8% (47) | 11.1% (2) | 0.155 | 3.15 |
| Dysgeusia | 20.2% (250) | 47.1% (8) | 0.013 | 3.50 |
| Dysosmia | 16.5% (203) | 29.4% (5) | 0.182 | 2.11 |
| Abdominal pain | 6.5% (79) | 14.3% (3) | 0.157 | 2.40 |
| Diarrhea | 14.8% (172) | 25.0% (5) | 0.206 | 1.92 |
| Bloody stool | 3.6% (44) | 10.5% (2) | 0.154 | 3.16 |

**Supplementary Table 2. COVID-19 severity and risk factors by stepwise logistic regression analysis**

| **Risk factor** | **Odds ratio** | **p value** | **Logistic regression**  **AIC=124.86** |
| --- | --- | --- | --- |
| BMI | 1.20 | 0.0068 |  |
| Cerebrovascular diseases history | 1.14 | 0.0293 |  |

BMI, Body mass index: AIC, Akaike’s information criterion
