# Supplementary figures and images for: Final Analysis of COVID-19 Patients With Inflammatory Bowel Disease in Japan (J-COSMOS): A Multicenter Registry Cohort Study
Source: Gastro Hep Adv. 2023 Jul 31;2(8):1056–65. doi: 10.1016/j.gastha.2023.07.017 (PMC11307685; doi:10.1016/j.gastha.2023.07.017)

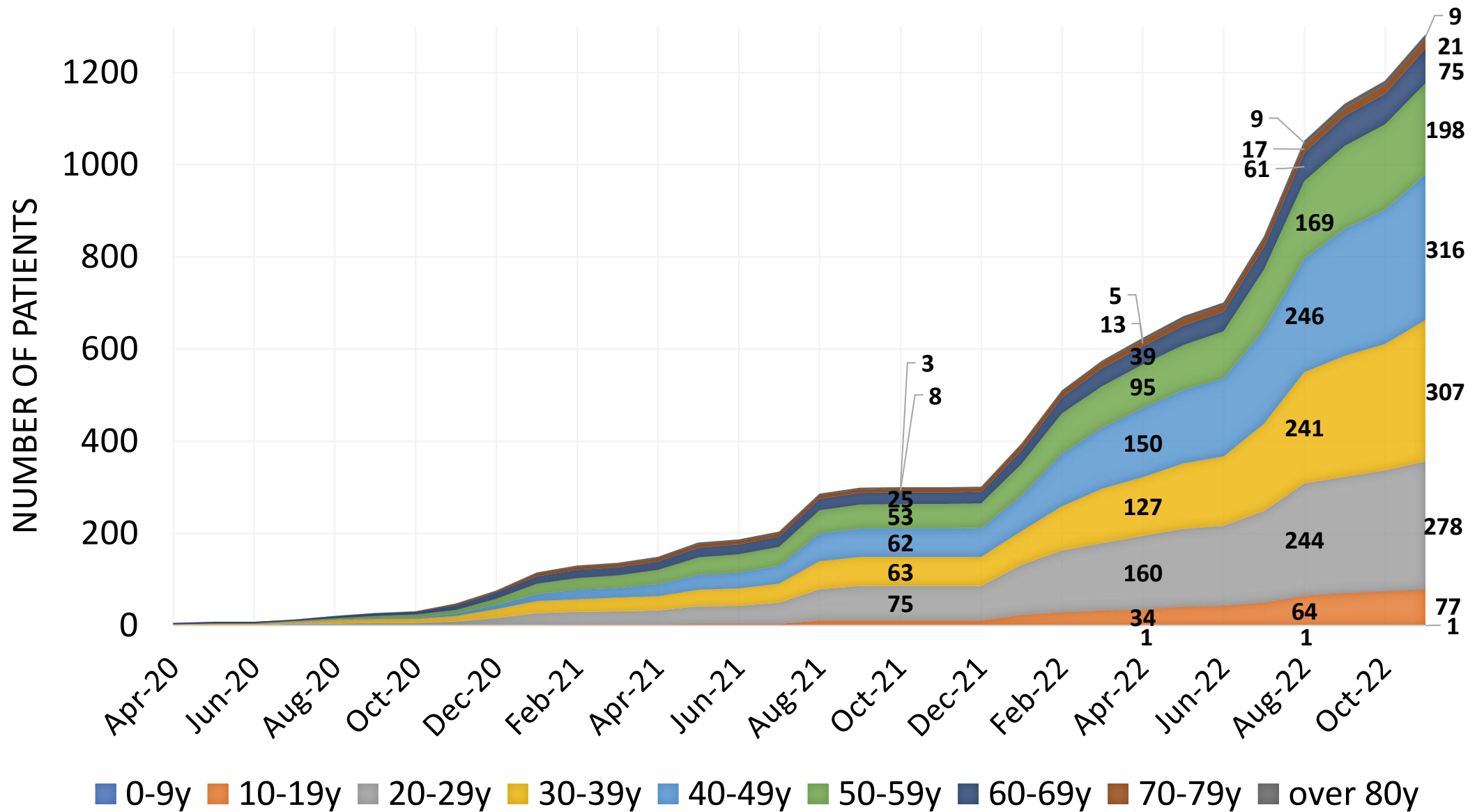

Supplementary Figure 1.

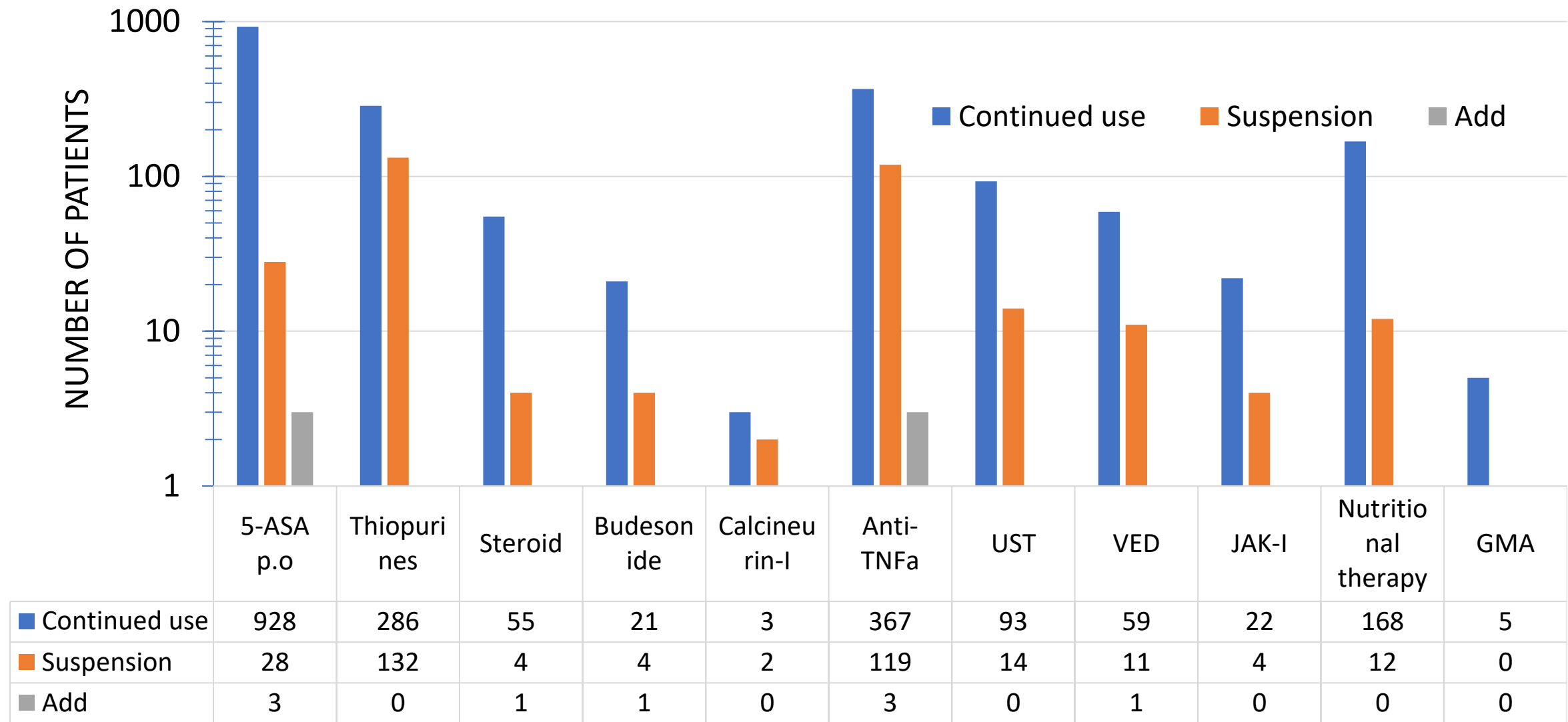

Supplementary Figure 2.

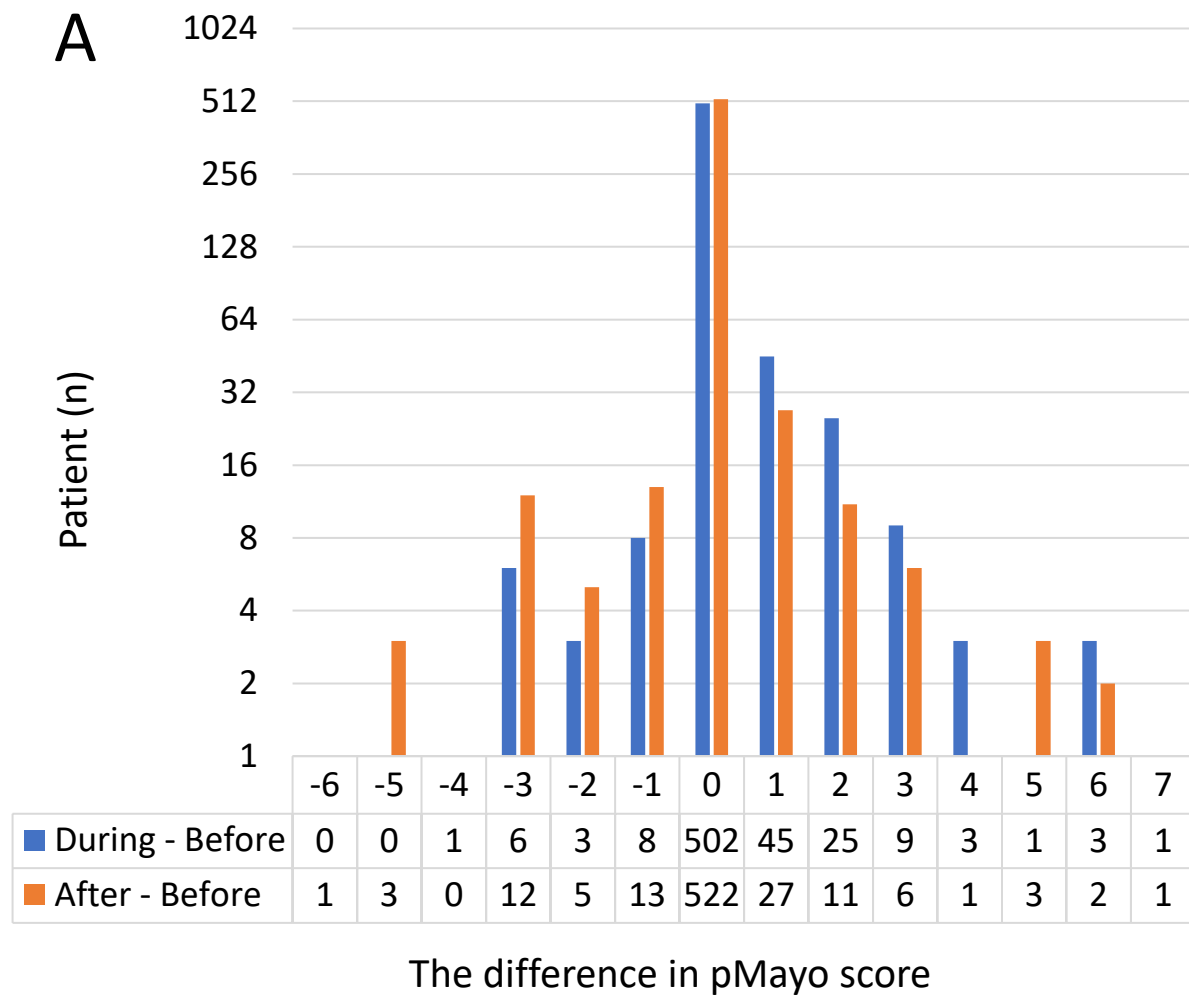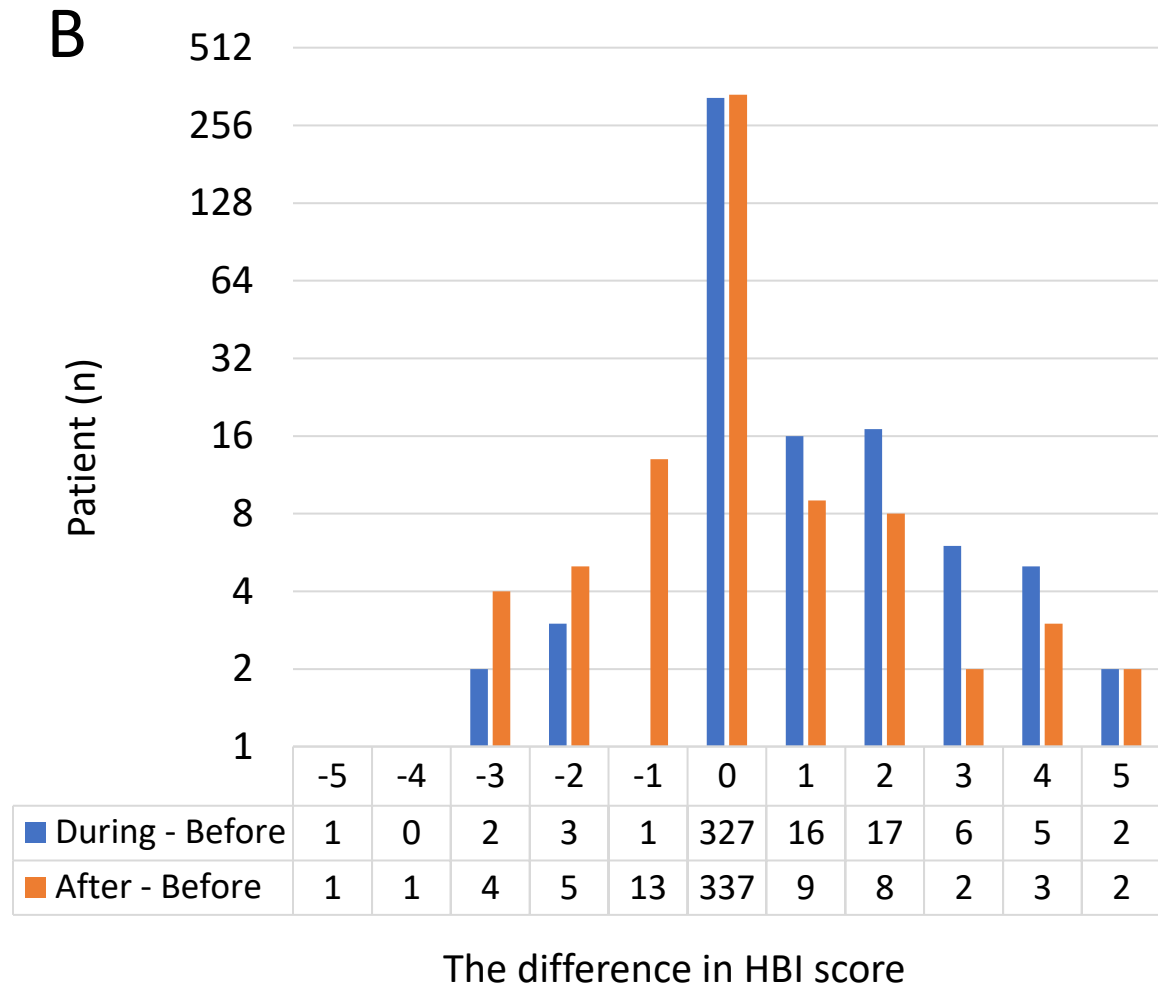

Supplementary Figure 3.

Supplement: Supplementary Figures JCOSMOS [file mmc2.pdf]
